# Symplectic superposition solutions for free in-plane vibration of orthotropic rectangular plates with general boundary conditions

**DOI:** 10.1038/s41598-023-29044-7

**Published:** 2023-02-14

**Authors:** Zhaoyang Hu, Jingyu Du, Mingfeng Liu, Yihao Li, Zixuan Wang, Xinran Zheng, Tinh Quoc Bui, Rui Li

**Affiliations:** 1 State Key Laboratory of Structural Analysis, Optimization and CAE Software for Industrial Equipment, Department of Engineering Mechanics, and International Research Center for Computational Mechanics, Dalian University of Technology, Dalian, 116024 China; 2 University of Michigan – Shanghai Jiao Tong University Joint Institute, Shanghai Jiao Tong University, Shanghai, 200240 China; 3 Duy Tan Research Institute for Computational Engineering (DTRICE), Duy Tan University, Ho Chi Minh City, 700000 Vietnam; 4 Faculty of Civil Engineering, Duy Tan University, Da Nang, 550000 Vietnam

**Keywords:** Mechanical engineering, Aerospace engineering

## Abstract

This work reports new analytic free in-plane vibration solutions for orthotropic non-Lévy-type rectangular plates, i.e., those without two opposite edges simply supported, by the symplectic superposition method (SSM), which has never been applied to in-plane elasticity problems in any existing works. Such analytic solutions are not accessible through conventional analytic methods as seeking analytic solutions that meet both the governing partial differential equations and various non-Lévy-type boundary conditions is an acknowledged challenge in mechanical analysis of plates. The clamped and free plates are considered as two most representative cases of non-Lévy-type plates. The SSM is implemented in the Hamiltonian system-based symplectic space, where the separation of variables and the symplectic eigen expansion prove to be well-grounded. These two mathematical treatments are adopted to first gain the analytic solutions of two elementary problems. The final analytic free in-plane vibration solutions are obtained by superposition of the two elementary problems. Comprehensive new natural frequencies and vibration modes are studied and validated by reference solutions from the finite element method or other approaches. The rigorous solution procedure, fast convergence, and highly accurate results render the present framework capable of serving as benchmarks for future comparison and applicable to analytic investigation of more plate problems.

## Introduction

Plate structures are widely used as fundamental engineering components in broad engineering fields including mechanical, aerospace, civil, and acoustical engineering. To realize structural safety designs, it is of significance to comprehensively investigate different dynamic behaviors of plates. During the last few decades, free vibration problems of plates have attracted considerable attention among researchers, and their efforts mainly focus on transverse vibration because transverse vibration corresponds to lower natural frequencies and is thus more likely to be excited. However, in-plane vibration does take place in some circumstances such as energy and sound transmission in build-up structures^1,2^, which demands further studies on the topic.

To provide a concise acquaintance of the progress in the field, representative works on free in-plane vibration of plates are reviewed. Bardell et al.^3^ utilized the Rayleigh–Ritz method to analyze free in-plane vibration of isotropic rectangular plates with a variety of different boundary conditions, which might be the first systematic investigation on the topic and hence provides important benchmarks for subsequent studies. Gorman^4,5^ employed the semi-inverse superposition method for a series of free in-plane vibration analyses, including isotropic and orthotropic rectangular plates. Du et al.^6^ investigated in-plane vibration of isotropic rectangular plates with elastically restrained edges via the improved Fourier series method. Using the same method, Zhang et al.^7^ studied in-plane vibration of orthotropic rectangular plates. Xing and Liu^8,9^ utilized the direct separation of variables to derive solutions for free in-plane vibration of isotropic and orthotropic rectangular plates with two opposite edges simply supported. Similarly, Wang et al.^10^ employed the iterative separation of variables to study free in-plane vibration of rectangular plates with homogeneous boundary conditions. With the aid of the separation of variables and hyperbolic function expansions, Deutsch and Eisenberger^11^ proposed analytic solutions on the free in-plane vibration of orthotropic square plates. Dozio^12^ presented a Ritz method with a set of trigonometric functions to investigate in-plane vibration of rectangular plates with non-uniform elastically restrained edges. Based on the same method, Dozio^13^ also investigated free in-plane vibration of single-layer and symmetrically laminated rectangular composite plates. Liu and Banerjee^14^ adopted the spectral dynamic stiffness method for in-plane vibration problems under plane stress and plane strain conditions, respectively. Liu et al.^15^ carried out free in-plane vibration analyses of plates in curvilinear domains by the differential quadrature hierarchical finite element method. Liu et al.^16^ investigated free in-plane vibration of arbitrarily shaped straight-sided quadrilateral and triangular plates based on the improved Fourier series method together with the coordinate transformation.

From the open literature, analytic solutions for free in-plane vibration of orthotropic rectangular plates with non-Lévy-type boundary conditions, i.e., those without two opposite edges simply supported, are still quite deficient, which motivates the present work. It is noteworthy that a symplectic superposition method (SSM) has been developed recently to deal with plate and shell problems involving out-of-plane deformation, including bending^17,18^, buckling^19,20^, and transverse vibration^21–23^. Such an analytic method is developed based on an elegant integration of the superposition technique and the symplectic approach^24,25^; it is not conducted in the Euclidean space but in the symplectic space where several important mathematical treatments, such as the separation of variables and the symplectic eigen expansion, prove to be valid. It is important to stress out that the SSM has never been applied to in-plane elasticity problems in any existing works.

This work aims at extending the SSM to study free in-plane vibration of non-Lévy-type rectangular plates, filling the gap in the aforementioned research field. The main novelty lies on yielding new analytic free in-plane vibration solutions by a rational and rigorous solution procedure of the SSM, without any assumption of solution forms, which distinguishes it from the conventional semi-inverse analytic methods and makes it possible to find analytic solutions that can satisfy both the governing partial differential equations and non-Lévy-type boundary conditions. In particular, two representative cases are considered, i.e., clamped plates and free plates. Comprehensive new analytic vibration results are presented including natural frequencies and vibration modes. Without loss of generality, both isotropic and orthotropic cases are taken into account. The new analytic results presented in this paper are well validated by reference solutions derived from the finite element method (FEM) through ABAQUS^26^ or other methods available in the open literature. This work not only provides benchmarks for free in-plane vibration studies but also extends the applicability of the SSM.

## Methods

### Governing equation for free in-plane vibration in the Hamiltonian system

The equilibrium equations for free in-plane vibration in the rectangular coordinate system *oxy* can be written as

1 $$\begin{gathered} \frac{{\partial \sigma_{y} }}{\partial y} + \frac{{\partial \tau_{xy} }}{\partial x} + \rho \omega^{2} v = 0 \hfill \\ \frac{{\partial \tau_{xy} }}{\partial y} + \frac{{\partial \sigma_{x} }}{\partial x} + \rho \omega^{2} u = 0 \hfill \\ \end{gathered}$$

where *u* and *v* are the in-plane modal displacements in *x* and *y* directions, respectively; $\sigma_{x}$ and $\sigma_{y}$ are the in-plane normal stresses in *x* and *y* directions, respectively; $\tau_{xy}$ is the shear stress; $\rho$ is the mass density; and $\omega$ is the natural frequency.

Without loss of generality, we first focus on orthotropic plates, and thus the in-plane normal and shear stresses in Eq. (1) are expressed as

2 $$\begin{gathered} \sigma_{x} = A_{11} \frac{\partial u}{{\partial x}} + A_{12} \frac{\partial v}{{\partial y}} \hfill \\ \sigma_{y} = A_{22} \frac{\partial v}{{\partial y}} + A_{21} \frac{\partial u}{{\partial x}} \hfill \\ \tau_{xy} = A_{66} \left( {\frac{\partial v}{{\partial x}} + \frac{\partial u}{{\partial y}}} \right) \hfill \\ \end{gathered}$$

where $A_{11} = {{E_{x} } \mathord{\left/ {\vphantom {{E_{x} } {\left( {1 - \nu_{x} \nu_{y} } \right)}}} \right. \kern-0pt} {\left( {1 - \nu_{x} \nu_{y} } \right)}}$, $A_{12} = {{\nu_{x} E_{x} } \mathord{\left/ {\vphantom {{\nu_{x} E_{x} } {\left( {1 - \nu_{x} \nu_{y} } \right)}}} \right. \kern-0pt} {\left( {1 - \nu_{x} \nu_{y} } \right)}}$, $A_{21} = {{\nu_{y} E_{y} } \mathord{\left/ {\vphantom {{\nu_{y} E_{y} } {\left( {1 - \nu_{x} \nu_{y} } \right)}}} \right. \kern-0pt} {\left( {1 - \nu_{x} \nu_{y} } \right)}}$, $A_{22} = {{E_{y} } \mathord{\left/ {\vphantom {{E_{y} } {\left( {1 - \nu_{x} \nu_{y} } \right)}}} \right. \kern-0pt} {\left( {1 - \nu_{x} \nu_{y} } \right)}}$, $A_{66} = G_{xy}$; $E_{x}$ and $E_{y}$ are Young's moduli in *x* and *y* directions, respectively; $\nu_{x}$ and $\nu_{y}$ are Poisson's ratios; and $G_{xy}$ is the shear modulus as expressed by $G_{xy} = {{\sqrt {E_{x} E_{y} } } \mathord{\left/ {\vphantom {{\sqrt {E_{x} E_{y} } } {\left[ {2\left( {1 + \sqrt {\nu_{x} \nu_{y} } } \right)} \right]}}} \right. \kern-0pt} {\left[ {2\left( {1 + \sqrt {\nu_{x} \nu_{y} } } \right)} \right]}}$. Note that the Betti principle $\nu_{x} E_{x} = \nu_{y} E_{y}$ gives $A_{12} = A_{21}$.

From the last two equations of Eq. (2), we respectively have

3 $$\frac{\partial v}{{\partial y}} = \frac{1}{{A_{22} }}\sigma_{y} - \frac{{A_{21} }}{{A_{22} }}\frac{\partial u}{{\partial x}}$$

and

4 $$\frac{\partial u}{{\partial y}} = \frac{1}{{A_{66} }}\tau_{xy} - \frac{\partial v}{{\partial x}}$$

From the first equation of Eq. (1), we have

5 $$\frac{{\partial \sigma_{y} }}{\partial y} = - \rho \omega^{2} v - \frac{{\partial \tau_{xy} }}{\partial x}$$

From the second equation of Eq. (1), together with the first equation of Eq. (2), yield

6 $$\frac{{\partial \tau_{xy} }}{\partial y} = - \rho \omega^{2} u - \frac{{A_{12} }}{{A_{22} }}\frac{{\partial \sigma_{y} }}{\partial x} + \left( {\frac{{A_{12} A_{21} }}{{A_{22} }} - A_{11} } \right)\frac{{\partial^{2} u}}{{\partial x^{2} }}$$

From Eqs. (3)–(6), the following matrix equation is obtained:

7 $$\frac{{\partial {\mathbf{Z}}}}{\partial y} = {\mathbf{HZ}}$$

where ${\mathbf{Z}} = \left[ {v,u,\sigma_{y} ,\tau_{xy} } \right]^{{\text{T}}}$, $${\mathbf{H}} = \left[ {\begin{array}{*{20}c} {\mathbf{F}} & {\mathbf{G}} \\ {\mathbf{Q}} & { - {\mathbf{F}}^{{\mathbf{T}}} } \\ \end{array} } \right]$$, with $${\mathbf{F}} = \left[ {\begin{array}{*{20}c} 0 & { - \left( {{{A_{21} } \mathord{\left/ {\vphantom {{A_{21} } {A_{22} }}} \right. \kern-0pt} {A_{22} }}} \right){\partial \mathord{\left/ {\vphantom {\partial {\partial x}}} \right. \kern-0pt} {\partial x}}} \\ { - {\partial \mathord{\left/ {\vphantom {\partial {\partial x}}} \right. \kern-0pt} {\partial x}}} & 0 \\ \end{array} } \right]$$,$${\mathbf{G}} = \left[ {\begin{array}{*{20}c} {{1 \mathord{\left/ {\vphantom {1 {A_{22} }}} \right. \kern-0pt} {A_{22} }}} & 0 \\ 0 & {{1 \mathord{\left/ {\vphantom {1 {A_{66} }}} \right. \kern-0pt} {A_{66} }}} \\ \end{array} } \right]$$, and $${\mathbf{Q}} = \left[ {\begin{array}{*{20}c} { - \rho \omega^{2} } & 0 \\ 0 & { - \rho \omega^{2} + \left( {{{A_{12} A_{21} } \mathord{\left/ {\vphantom {{A_{12} A_{21} } {A_{22} }}} \right. \kern-0pt} {A_{22} }} - A_{11} } \right){{\partial^{2} } \mathord{\left/ {\vphantom {{\partial^{2} } {\partial x^{2} }}} \right. \kern-0pt} {\partial x^{2} }}} \\ \end{array} } \right]$$. The Hamiltonian operator matrix ${\mathbf{H}}$ meets ${\mathbf{H}}^{{\text{T}}} = {\mathbf{JHJ}}$, with $${\mathbf{J}} = \left[ {\begin{array}{*{20}c} 0 & {{\mathbf{I}}_{2} } \\ { - {\mathbf{I}}_{2} } & 0 \\ \end{array} } \right]$$, where ${\mathbf{I}}_{2}$ is the $2 \times 2$ unit matrix^24^. Thus, Eq. (7) serves as the governing equation for free in-plane vibration in the Hamiltonian system.

### New analytic free in-plane vibration solutions of rectangular plates

For convenience, “clamped” and “free” boundary conditions are denoted by their abbreviations “C” and “F”, respectively. Two kinds of “simply supported” boundary conditions mentioned in Refs.^8,9^ are also adopted, which are denoted by “SS1” and “SS2”, respectively. In particular, at $x = {0}$ and $x = a$, we have $u = 0$ and $v = 0$ for C edges, $\sigma_{x} = 0$ and $\tau_{xy} = 0$ for F edges, $v = 0$ and $\sigma_{x} = 0$ for SS1 edges, and $u = 0$ and $\tau_{xy} = 0$ for SS2 edges; at $y = {0}$ and $y = b$, we have $u = 0$ and $v = 0$ for C edges, $\sigma_{y} = 0$ and $\tau_{xy} = 0$ for F edges, $u = 0$ and $\sigma_{y} = 0$ for SS1 edges, and $v = 0$ and $\tau_{xy} = 0$ for SS2 edges.

With the Hamiltonian system-based governing equation, i.e., Eq. (7), we implement the SSM herein to obtain new analytic free in-plane vibration solutions of C–C–C–C and F–F–F–F rectangular plates. In Section "Basic symplectic analytic solutions of two kinds of elementary problems", the basic analytic solutions of the elementary problems are first gained based on the mathematical techniques in the symplectic space. Then, by superposition of the elementary problems’ solutions, the final analytic free in-plane vibration solutions of C–C–C–C and F–F–F–F plates are given in Sections "Symplectic superposition solutions of C–C–C–C plates" and "Symplectic superposition solutions of F–F–F–F plates", respectively.

#### Basic symplectic analytic solutions of two kinds of elementary problems

The separation of variables, which has been proven to be valid in the symplectic space^24^, is utilized to solve Eq. (7), leading to

8 $${\mathbf{HX}}\left( x \right) = \mu {\mathbf{X}}\left( x \right)$$

9 $$\frac{{{\text{d}}Y\left( y \right)}}{{{\text{d}}y}} = \mu Y\left( y \right)$$

where $\mu$ is the eigenvalue and ${\mathbf{X}}\left( x \right){ = }\left[ {v\left( x \right),u\left( x \right),\sigma_{y} \left( x \right),\tau_{xy} \left( x \right)} \right]^{{\text{T}}}$ is the corresponding eigenvector. Equating the characteristic equation of Eq. (8) to zero gives the eigensolution of ${\mathbf{X}}\left( x \right)$^27^:

10 $$\begin{gathered} v\left( x \right) = A_{1} \cosh \left( {\lambda_{1} x} \right) + B_{1} \cosh \left( {\lambda_{2} x} \right) + C_{1} \sinh \left( {\lambda_{1} x} \right) + D_{1} \sinh \left( {\lambda_{2} x} \right) \hfill \\ u\left( x \right) = A_{2} \cosh \left( {\lambda_{1} x} \right) + B_{2} \cosh \left( {\lambda_{2} x} \right) + C_{2} \sinh \left( {\lambda_{1} x} \right) + D_{2} \sinh \left( {\lambda_{2} x} \right) \hfill \\ \sigma_{y} \left( x \right) = A_{3} \cosh \left( {\lambda_{1} x} \right) + B_{3} \cosh \left( {\lambda_{2} x} \right) + C_{3} \sinh \left( {\lambda_{1} x} \right) + D_{3} \sinh \left( {\lambda_{2} x} \right) \hfill \\ \tau_{xy} \left( x \right) = A_{4} \cosh \left( {\lambda_{1} x} \right) + B_{4} \cosh \left( {\lambda_{2} x} \right) + C_{4} \sinh \left( {\lambda_{1} x} \right) + D_{4} \sinh \left( {\lambda_{2} x} \right) \hfill \\ \end{gathered}$$

where $\lambda_{1}$ and $\lambda_{2}$ are expressed as

11 $$\begin{gathered} \lambda_{1} = \sqrt { - \frac{{\gamma_{1} + \sqrt {\gamma_{1}^{2} - \gamma_{2} } }}{{2\eta_{1} }}} \hfill \\ \lambda_{2} = \sqrt { - \frac{{\gamma_{1} - \sqrt {\gamma_{1}^{2} - \gamma_{2} } }}{{2\eta_{1} }}} \hfill \\ \end{gathered}$$

with $\gamma_{1} = \left( {1 + \eta_{1} } \right)R^{2} + \left( {1 + \eta_{1} \eta_{2} - \eta_{3}^{2} } \right)\mu^{2}$, $\gamma_{2} = 4\eta_{1} \left( {R^{2} + \mu^{2} } \right)\left( {R^{2} + \eta_{2} \mu^{2} } \right)$, $\eta_{1} = {{A_{11} } \mathord{\left/ {\vphantom {{A_{11} } {A_{66} }}} \right. \kern-0pt} {A_{66} }}$, $\eta_{2} = {{A_{22} } \mathord{\left/ {\vphantom {{A_{22} } {A_{66} }}} \right. \kern-0pt} {A_{66} }}$, $\eta_{3} = {{\left( {A_{12} + A_{66} } \right)} \mathord{\left/ {\vphantom {{\left( {A_{12} + A_{66} } \right)} {A_{66} }}} \right. \kern-0pt} {A_{66} }}$, and $R = \omega \sqrt {{\rho \mathord{\left/ {\vphantom {\rho {A_{66} }}} \right. \kern-0pt} {A_{66} }}}$. Substituting Eq. (10) into Eq. (8) yields the relationships among the constant coefficients $A_{1 \sim 4}$, $B_{1 \sim 4}$, $C_{1 \sim 4}$, and $D_{1 \sim 4}$: $A_{2} = - k_{1} C_{1}$, $B_{2} = - k_{2} D_{1}$, $C_{2} = - k_{1} A_{1}$, $D_{2} = - k_{2} B_{1}$, $A_{3} = k_{3} A_{1}$, $B_{3} = k_{4} B_{1}$, $C_{3} = k_{3} C_{1}$, $D_{3} = k_{4} D_{1}$, $A_{4} = - k_{5} C_{1}$, $B_{4} = - k_{6} D_{1}$, $C_{4} = - k_{5} A_{1}$, and $D_{4} = - k_{6} B_{1}$, such that only $A_{1}$, $B_{1}$, $C_{1}$, and $D_{1}$ are independent, which are to be determined by the boundary conditions at $x = {0}$ and $x = a$. Detailed expressions of $k_{1 \sim 6}$ are shown in Appendix 1.

To obtain the analytic free in-plane vibration solutions of C–C–C–C and F–F–F–F plates, two kinds of elementary problems shall be analytically solved, as elaborated in the following.

##### Plate with two opposite SS1 edges

The boundary conditions of a plate with the pair of parallel SS1 edges at $x = {0}$ and $x = a$ are written as

12 $$\begin{gathered} \left. v \right|_{x = 0,a} = 0 \hfill \\ \left. {\sigma_{x} } \right|_{x = 0,a} = 0 \hfill \\ \end{gathered}$$

Substituting Eq. (10) and the first equation of Eq. (2) into Eq. (12), for a non-trivial solution, we have $\sinh \left( {\lambda_{1} a} \right)\sinh \left( {\lambda_{2} a} \right) = 0$, which gives $\lambda_{1} = \lambda_{2} = \pm \alpha_{n} {\text{I}}$, with $\alpha_{n} = {{n\pi } \mathord{\left/ {\vphantom {{n\pi } a}} \right. \kern-0pt} a}$ ($n = 1,2,3, \ldots$) and ${\text{I}}$ being the imaginary unit. Under Eq. (11), the eigenvalues are obtained as

13 $$\begin{gathered} \mu_{1n} = \sqrt { - \frac{{\gamma_{3} + \sqrt {\gamma_{3}^{2} - \gamma_{4} } }}{{2\eta_{2} }}} \hfill \\ \mu_{2n} = - \mu_{1n} \hfill \\ \mu_{3n} = \sqrt { - \frac{{\gamma_{3} - \sqrt {\gamma_{3}^{2} - \gamma_{4} } }}{{2\eta_{2} }}} \hfill \\ \mu_{4n} = - \mu_{3n} \hfill \\ \end{gathered}$$

where $\gamma_{3} = \left( {1 + \eta_{2} } \right)R^{2} - \left( {1 + \eta_{1} \eta_{2} - \eta_{3}^{2} } \right)\alpha_{n}^{2}$ and $\gamma_{4} = 4\eta_{2} \left( {\alpha_{n}^{2} - R^{2} } \right)\left( {\eta_{1} \alpha_{n}^{2} - R^{2} } \right)$, and their corresponding eigenvectors are

14 $$\begin{gathered} {\mathbf{X}}_{1n} \left( x \right) = \left[ {\sin \left( {\alpha_{n} x} \right), - k_{1} \left( {\mu_{1n} } \right)\cos \left( {\alpha_{n} x} \right),k_{3} \left( {\mu_{1n} } \right)\sin \left( {\alpha_{n} x} \right), - k_{5} \left( {\mu_{1n} } \right)\cos \left( {\alpha_{n} x} \right)} \right]^{{\text{T}}} \hfill \\ {\mathbf{X}}_{2n} \left( x \right) = \left[ {\sin \left( {\alpha_{n} x} \right), - k_{1} \left( {\mu_{{{2}n}} } \right)\cos \left( {\alpha_{n} x} \right),k_{3} \left( {\mu_{{{2}n}} } \right)\sin \left( {\alpha_{n} x} \right), - k_{5} \left( {\mu_{{{2}n}} } \right)\cos \left( {\alpha_{n} x} \right)} \right]^{{\text{T}}} \hfill \\ {\mathbf{X}}_{3n} \left( x \right) = \left[ {\sin \left( {\alpha_{n} x} \right), - k_{2} \left( {\mu_{{{3}n}} } \right)\cos \left( {\alpha_{n} x} \right),k_{4} \left( {\mu_{{{3}n}} } \right)\sin \left( {\alpha_{n} x} \right), - k_{6} \left( {\mu_{{{3}n}} } \right)\cos \left( {\alpha_{n} x} \right)} \right]^{{\text{T}}} \hfill \\ {\mathbf{X}}_{4n} \left( x \right) = \left[ {\sin \left( {\alpha_{n} x} \right), - k_{2} \left( {\mu_{{{4}n}} } \right)\cos \left( {\alpha_{n} x} \right),k_{4} \left( {\mu_{{{4}n}} } \right)\sin \left( {\alpha_{n} x} \right), - k_{6} \left( {\mu_{{{4}n}} } \right)\cos \left( {\alpha_{n} x} \right)} \right]^{{\text{T}}} \hfill \\ \end{gathered}$$

There also exists a special case when $n = 0$, which corresponds to the constant eigenvalues $\mu^{\prime}_{1} = {\text{I}}R$ and $\mu^{\prime}_{2} = - {\text{I}}R$, and the constant eigenvectors ${\mathbf{X^{\prime}}}_{1} \left( x \right) = \left[ {0,1,0,{{k_{5} \left( {\mu^{\prime}_{1} } \right)} \mathord{\left/ {\vphantom {{k_{5} \left( {\mu^{\prime}_{1} } \right)} {k_{1} }}} \right. \kern-0pt} {k_{1} }}\left( {\mu^{\prime}_{1} } \right)} \right]^{{\text{T}}} \cos \left( {\alpha_{n} x} \right)$ and ${\mathbf{X^{\prime}}}_{2} \left( x \right) = \left[ {0,1,0,{{k_{5} \left( {\mu^{\prime}_{{2}} } \right)} \mathord{\left/ {\vphantom {{k_{5} \left( {\mu^{\prime}_{{2}} } \right)} {k_{1} \left( {\mu^{\prime}_{{2}} } \right)}}} \right. \kern-0pt} {k_{1} \left( {\mu^{\prime}_{{2}} } \right)}}} \right]^{{\text{T}}} \cos \left( {\alpha_{n} x} \right)$. Based on the symplectic eigen expansion^24^, the state vector ${\mathbf{Z}}$ is expressed as

15 $${\mathbf{Z}} = \;f^{\prime}_{1} e^{{\mu^{\prime}_{1} y}} {\mathbf{X^{\prime}}}_{1} + f^{\prime}_{2} e^{{\mu^{\prime}_{2} y}} {\mathbf{X^{\prime}}}_{2} + \sum\limits_{n = 1,2,3, \cdots }^{\infty } {\left( {f_{1n} e^{{\mu_{1n} y}} {\mathbf{X}}_{1n} + f_{2n} e^{{\mu_{2n} y}} {\mathbf{X}}_{2n} + f_{3n} e^{{\mu_{3n} y}} {\mathbf{X}}_{3n} + f_{4n} e^{{\mu_{4n} y}} {\mathbf{X}}_{4n} } \right)}$$

where $f^{\prime}_{1}$, $f^{\prime}_{2}$, $f_{1n}$, $f_{2n}$, $f_{3n}$, and $f_{4n}$ are the constant coefficients to be determined by the boundary conditions imposed at $y = {0}$ and $y = b$.

##### Plate with two opposite SS2 edges

The boundary conditions of a plate with the pair of parallel SS2 edges at $x = {0}$ and $x = a$ are written as

16 $$\begin{gathered} \left. u \right|_{x = 0,a} = 0 \hfill \\ \left. {\tau_{xy} } \right|_{x = 0,a} = 0 \hfill \\ \end{gathered}$$

Substituting Eq. (10) into Eq. (16), for a nontrivial solution, we have $\sinh \left( {\lambda_{1} a} \right)\sinh \left( {\lambda_{2} a} \right) = 0$, which gives $\lambda_{1} = \lambda_{2} = \pm \alpha_{n} {\text{I}}$. The eigenvalues are the same as those in Eq. (13) while the corresponding eigenvectors are

17 $$\begin{gathered} {\mathbf{X}}_{1n} \left( x \right) = \left[ {\cos \left( {\alpha_{n} x} \right), - k_{1} \left( {\mu_{1n} } \right)\sin \left( {\alpha_{n} x} \right),k_{3} \left( {\mu_{1n} } \right)\cos \left( {\alpha_{n} x} \right), - k_{5} \left( {\mu_{1n} } \right)\sin \left( {\alpha_{n} x} \right)} \right]^{{\text{T}}} \hfill \\ {\mathbf{X}}_{2n} \left( x \right) = \left[ {\cos \left( {\alpha_{n} x} \right), - k_{1} \left( {\mu_{{{2}n}} } \right)\sin \left( {\alpha_{n} x} \right),k_{3} \left( {\mu_{{{2}n}} } \right)\cos \left( {\alpha_{n} x} \right), - k_{5} \left( {\mu_{{{2}n}} } \right)\sin \left( {\alpha_{n} x} \right)} \right]^{{\text{T}}} \hfill \\ {\mathbf{X}}_{3n} \left( x \right) = \left[ {\cos \left( {\alpha_{n} x} \right), - k_{2} \left( {\mu_{{{3}n}} } \right)\sin \left( {\alpha_{n} x} \right),k_{4} \left( {\mu_{{{3}n}} } \right)\cos \left( {\alpha_{n} x} \right), - k_{6} \left( {\mu_{{{3}n}} } \right)\sin \left( {\alpha_{n} x} \right)} \right]^{{\text{T}}} \hfill \\ {\mathbf{X}}_{4n} \left( x \right) = \left[ {\cos \left( {\alpha_{n} x} \right), - k_{2} \left( {\mu_{{{4}n}} } \right)\sin \left( {\alpha_{n} x} \right),k_{4} \left( {\mu_{{{4}n}} } \right)\cos \left( {\alpha_{n} x} \right), - k_{6} \left( {\mu_{{{4}n}} } \right)\sin \left( {\alpha_{n} x} \right)} \right]^{{\text{T}}} \hfill \\ \end{gathered}$$

Based on the symplectic eigen expansion, the state vector ${\mathbf{Z}}$ is expressed as

18 $${\mathbf{Z}} = \;\sum\limits_{n = 1,2,3, \cdots }^{\infty } {\left( {f_{1n} e^{{\mu_{1n} y}} {\mathbf{X}}_{1n} + f_{2n} e^{{\mu_{2n} y}} {\mathbf{X}}_{2n} + f_{3n} e^{{\mu_{3n} y}} {\mathbf{X}}_{3n} + f_{4n} e^{{\mu_{4n} y}} {\mathbf{X}}_{4n} } \right)}$$

It should be noted that Eqs. (15) and (18) serve as the analytic solutions for the free in-plane vibration of Lévy-type plates, based on which the frequency results of some representative Lévy-type plates are tabulated in Appendix 2.

#### Symplectic superposition solutions of C–C–C–C plates

**Figure 1:**
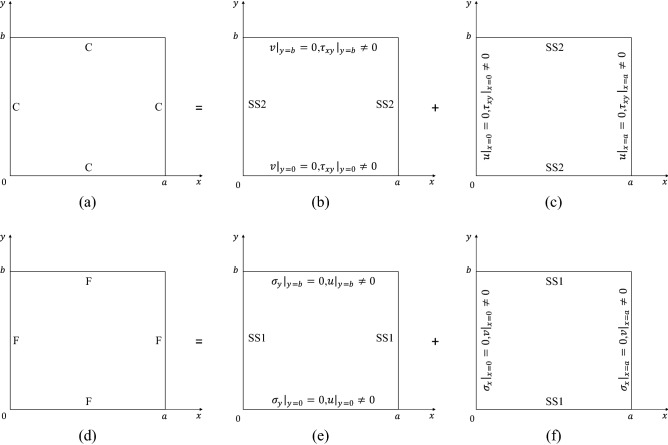
Symplectic superposition for free in-plane vibration. C–C–C–C plates: (**a**) the original problem, (**b**) the first elementary problem, and (**c**) the second elementary problem. F–F–F–F plates: (**d**) the original problem, (**e**) the first elementary problem, and (**f**) the second elementary problem.

For a C–C-C–C plate shown in Fig. 1(a), the problem is divided into two elementary problems shown in Fig. 1b,c, respectively. In the first elementary problem, the plate is treated as an SS2–SS2–SS2–SS2 plate with imposed nonzero shear stresses at $y = {0}$ and $y = b$, expressed by $\left. {\tau_{xy} } \right|_{y = 0} = \sum\nolimits_{n = 1,2,3, \cdots }^{\infty } {E_{n} \sin \left( {\alpha_{n} x} \right)}$ and $\left. {\tau_{xy} } \right|_{y = b} = \sum\nolimits_{n = 1,2,3, \cdots }^{\infty } {F_{n} \sin \left( {\alpha_{n} x} \right)}$, respectively. In the second elementary problem, the plate is also treated as an SS2–SS2–SS2–SS2 plate with imposed nonzero shear stresses at $x = {0}$ and $x = a$, expressed by $\left. {\tau_{xy} } \right|_{x = 0} = \sum\nolimits_{n = 1,2,3, \cdots }^{\infty } {G_{n} \sin \left( {\beta_{n} y} \right)}$ and $\left. {\tau_{xy} } \right|_{x = a} = \sum\nolimits_{n = 1,2,3, \cdots }^{\infty } {H_{n} \sin \left( {\beta_{n} y} \right)}$, respectively. Here, $\beta_{n} = {{n\pi } \mathord{\left/ {\vphantom {{n\pi } b}} \right. \kern-0pt} b}$; $E_{n}$, $F_{n}$, $G_{n}$, and $H_{n}$ are the expansion coefficients. The analytic solutions of the two elementary problems are obtained by substituting Eq. (18) into the boundary conditions at $y = {0}$ and $y = b$ of the first elementary problem and the boundary conditions at $x = {0}$ and $x = a$ of the second elementary problem by aid of coordinate exchange, respectively.

For the first elementary problem, the dimensionless modal displacements $v_{1} \left( {\overline{x},\overline{y}} \right)$ and $u_{1} \left( {\overline{x},\overline{y}} \right)$ are obtained as

19 $$\begin{gathered} \frac{{v_{1} \left( {\overline{x},\overline{y}} \right)}}{a} = \sum\limits_{n = 1,2,3, \cdot \cdot \cdot }^{\infty } {\frac{{\cos \left( {n\pi \overline{x}} \right)}}{{n\pi \chi_{n} \eta_{3} }}\frac{{\xi_{1n} \xi_{2n} }}{{\left( {\xi_{1n} - \xi_{2n} } \right)}}} \\ \times \left\{ {\overline{E}_{n} \left\{ {{\text{csch}} \left( {\phi \varepsilon_{1n} } \right)\sinh \left[ {\phi \varepsilon_{1n} \left( {\overline{y} - 1} \right)} \right] - {\text{csch}} \left( {\phi \varepsilon_{2n} } \right)\sinh \left[ {\phi \varepsilon_{2n} \left( {\overline{y} - 1} \right)} \right]} \right\}} \right. \\ \left. { + \overline{F}_{n} \left[ {{\text{csch}} \left( {\phi \varepsilon_{1n} } \right)\sinh \left( {\phi \varepsilon_{1n} \overline{y}} \right) - {\text{csch}} \left( {\phi \varepsilon_{2n} } \right)\sinh \left( {\phi \varepsilon_{2n} \overline{y}} \right)} \right]} \right\} \\ \end{gathered}$$

and

20 $$\begin{gathered} \frac{{u_{1} \left( {\overline{x},\overline{y}} \right)}}{a} = \sum\limits_{n = 1,2,3, \ldots }^{\infty } {\frac{{\sin \left( {n\pi \overline{x}} \right)}}{{\chi_{n} \left( {\xi_{1n} - \xi_{2n} } \right)}}} \\ \times \left\{ {\overline{E}_{n} \left\{ {\xi_{1n} \varepsilon_{2n} {\text{csch}} \left( {\phi \varepsilon_{2n} } \right)\cosh \left[ {\phi \varepsilon_{2n} \left( {\overline{y} - 1} \right)} \right] - \xi_{2n} \varepsilon_{1n} {\text{csch}} \left( {\phi \varepsilon_{1n} } \right)\cosh \left[ {\phi \varepsilon_{1n} \left( {\overline{y} - 1} \right)} \right]} \right\}} \right. \\ \left. { + \overline{F}_{n} \left[ {\xi_{2n} \varepsilon_{1n} {\text{csch}} \left( {\phi \varepsilon_{1n} } \right)\cosh \left( {\phi \varepsilon_{1n} \overline{y}} \right) - \xi_{1n} \varepsilon_{2n} {\text{csch}} \left( {\phi \varepsilon_{2n} } \right)\cosh \left( {\phi \varepsilon_{2n} \overline{y}} \right)} \right]} \right\} \\ \end{gathered}$$

where $$\overline{x} = {x \mathord{\left/ {\vphantom {x a}} \right. \kern-0pt} a}$$, $$\overline{y} = {y \mathord{\left/ {\vphantom {y b}} \right. \kern-0pt} b}$$, $$\phi = {b \mathord{\left/ {\vphantom {b a}} \right. \kern-0pt} a}$$, $$\varepsilon_{1n} = a\mu_{1n}$$, $$\varepsilon_{2n} = a\mu_{3n}$$, $$\chi_{n} = R^{2} a^{2} + \eta_{1} n^{2} \pi^{2}$$, $$\xi_{1n} = \chi_{n} + \varepsilon_{1n}^{2}$$, $$\xi_{2n} = \chi_{n} + \varepsilon_{2n}^{2}$$, $$\overline{E}_{n} = {{E_{n} } \mathord{\left/ {\vphantom {{E_{n} } {A_{66} }}} \right. \kern-0pt} {A_{66} }}$$, and $$\overline{F}_{n} = {{F_{n} } \mathord{\left/ {\vphantom {{F_{n} } {A_{66} }}} \right. \kern-0pt} {A_{66} }}$$.

Due to the similarity of two elementary problems for a C–C–C–C plate, using coordinate exchange, i.e., exchanging $x$ and $y$, $a$ and $b$, and replacing $E_{n}$ with $G_{n}$, and $F_{n}$ with $H_{n}$, the dimensionless modal displacements $u_{2} \left( {\overline{x},\overline{y}} \right)$ and $v_{2} \left( {\overline{x},\overline{y}} \right)$ can be readily obtained from $v_{1} \left( {\overline{x},\overline{y}} \right)$ and $u_{1} \left( {\overline{x},\overline{y}} \right)$, respectively.

For a C–C–C–C plate, zero in-plane modal displacement constraints must be ensured at $x = {0}$, $x = a$, $y = 0$, and $y = b$, i.e.,

21 $$\begin{gathered} \sum\limits_{i = 1}^{2} {\left. {v_{i} } \right|_{x = 0,a} } = 0 \hfill \\ \sum\limits_{i = 1}^{2} {\left. {u_{i} } \right|_{y = 0,b} } = 0 \hfill \\ \end{gathered}$$

Under Eq. (21), four sets of linear equations are generated. Equating the determinant of the coefficient matrix with respect to $E_{n}$, $F_{n}$, $G_{n}$, and $H_{n}$ to zero yields nontrivial natural frequencies of a C–C–C–C plate under in-plane vibration. Taking a natural frequency back into Eq. (21), $E_{n}$, $F_{n}$, $G_{n}$, and $H_{n}$ are given. The corresponding vibration modes are obtained by substituting such coefficients into the summation of the in-plane modal displacement solutions of the elementary problems.

#### Symplectic superposition solutions of F–F–F–F plates

For an F-F-F-F plate shown in Fig. 1d, the problem is divided into two elementary problems shown in Fig. 1e,f, respectively. In the first elementary problem, the plate is treated as an SS1–SS1–SS1–SS1 plate with imposed nonzero in-plane modal displacements in *x* direction at $y = {0}$ and $y = b$, expressed by $\left. u \right|_{y = 0} = \sum\nolimits_{n = 0,1,2, \cdots }^{\infty } {E_{n} \cos \left( {\alpha_{n} x} \right)}$ and $\left. u \right|_{y = b} = \sum\nolimits_{n = 0,1,2, \cdots }^{\infty } {F_{n} \cos \left( {\alpha_{n} x} \right)}$, respectively. In the second elementary problem, the plate is also treated as an SS1–SS1–SS1–SS1 plate with imposed nonzero in-plane modal displacements in *y* direction at $x = {0}$ and $x = a$, expressed by $\left. v \right|_{x = 0} = \sum\nolimits_{n = 0,1,2, \cdots }^{\infty } {G_{n} \cos \left( {\beta_{n} y} \right)}$ and $\left. v \right|_{x = a} = \sum\nolimits_{n = 0,1,2, \cdots }^{\infty } {H_{n} \cos \left( {\beta_{n} y} \right)}$, respectively. The analytic solutions of the two elementary problems are obtained by substituting Eq. (15) into the boundary conditions at $y = {0}$ and $y = b$ of the first elementary problem and the boundary conditions at $x = {0}$ and $x = a$ of the second elementary problem by aid of coordinate exchange, respectively.

For the first elementary problem, the dimensionless modal displacements $v_{1} \left( {\overline{x},\overline{y}} \right)$ and $u_{1} \left( {\overline{x},\overline{y}} \right)$ are obtained as

22 $$\begin{gathered} \frac{{v_{1} \left( {\overline{x},\overline{y}} \right)}}{a} = \sum\limits_{n = 1,2,3, \cdot \cdot \cdot }^{\infty } {\frac{{\sin \left( {n\pi \overline{x}} \right)}}{{n\pi \eta_{2} \eta_{3} \varepsilon_{1n} \varepsilon_{2n} \left( {\xi_{1n} - \xi_{2n} } \right)}}} \\ \times \left\{ {\tilde{E}_{n} \left\{ {\varepsilon_{1n} \xi_{2n} \left[ {\eta_{2} \xi_{1n} - \left( {\eta_{3}^{2} - \eta_{3} } \right)n^{2} \pi^{2} } \right]{\text{csch}} \left( {\phi \varepsilon_{2n} } \right)\cosh \left[ {\phi \varepsilon_{2n} \left( {\overline{y} - 1} \right)} \right]} \right.} \right. \\ \left. { - \varepsilon_{2n} \xi_{1n} \left[ {\eta_{2} \xi_{2n} - \left( {\eta_{3}^{2} - \eta_{3} } \right)n^{2} \pi^{2} } \right]{\text{csch}} \left( {\phi \varepsilon_{1n} } \right)\cosh \left[ {\phi \varepsilon_{1n} \left( {\overline{y} - 1} \right)} \right]} \right\} \\ + \tilde{F}_{n} \left\{ {\varepsilon_{2n} \xi_{1n} \left[ {\eta_{2} \xi_{2n} - \left( {\eta_{3}^{2} - \eta_{3} } \right)n^{2} \pi^{2} } \right]{\text{csch}} \left( {\phi \varepsilon_{1n} } \right)\cosh \left( {\phi \varepsilon_{1n} \overline{y}} \right)} \right. \\ \left. {\left. { - \varepsilon_{1n} \xi_{2n} \left[ {\eta_{2} \xi_{1n} - \left( {\eta_{3}^{2} - \eta_{3} } \right)n^{2} \pi^{2} } \right]{\text{csch}} \left( {\phi \varepsilon_{2n} } \right)\cosh \left( {\phi \varepsilon_{2n} \overline{y}} \right)} \right\}} \right\} \\ \end{gathered}$$

and

23 $$\begin{gathered} \frac{{u_{1} \left( {\overline{x},\overline{y}} \right)}}{a} = \tilde{F}_{0} {\text{csch}} \left( {\phi \varepsilon_{0} } \right)\sinh \left( {\phi \varepsilon_{0} \overline{y}} \right) - \tilde{E}_{0} {\text{csch}} \left( {\phi \varepsilon_{0} } \right)\sinh \left[ {\phi \varepsilon_{0} \left( {\overline{y} - 1} \right)} \right] + \sum\limits_{n = 1,2,3, \ldots }^{\infty } {\frac{{\cos \left( {n\pi \overline{x}} \right)}}{{\eta_{2} \left( {\xi_{1n} - \xi_{2n} } \right)}}} \\ \times \left\{ {\tilde{E}_{n} \left\{ {\left[ {\eta_{2} \xi_{1n} - \left( {\eta_{3}^{2} - \eta_{3} } \right)n^{2} \pi^{2} } \right]{\text{csch}} \left( {\phi \varepsilon_{2n} } \right)\sinh \left[ {\phi \varepsilon_{2n} \left( {\overline{y} - 1} \right)} \right]} \right.} \right. \\ \left. { - \left[ {\eta_{2} \xi_{2n} - \left( {\eta_{3}^{2} - \eta_{3} } \right)n^{2} \pi^{2} } \right]{\text{csch}} \left( {\phi \varepsilon_{1n} } \right)\sinh \left[ {\phi \varepsilon_{1n} \left( {\overline{y} - 1} \right)} \right]} \right\} \\ + \tilde{F}_{n} \left\{ {\varepsilon_{1n} \xi_{2n} \left[ {\eta_{2} \xi_{1n} - \left( {\eta_{3}^{2} - \eta_{3} } \right)n^{2} \pi^{2} } \right]{\text{csch}} \left( {\phi \varepsilon_{2n} } \right)\sinh \left( {\phi \varepsilon_{2n} \overline{y}} \right)} \right. \\ \left. {\left. { - \varepsilon_{2n} \xi_{1n} \left[ {\eta_{2} \xi_{2n} - \left( {\eta_{3}^{2} - \eta_{3} } \right)n^{2} \pi^{2} } \right]{\text{csch}} \left( {\phi \varepsilon_{1n} } \right)\sinh \left( {\phi \varepsilon_{1n} \overline{y}} \right)} \right\}} \right\} \\ \end{gathered}$$

where $$\varepsilon_{0} = a\mu^{\prime}_{1}$$, $$\tilde{E}_{0} = {{E_{0} } \mathord{\left/ {\vphantom {{E_{0} } a}} \right. \kern-0pt} a}$$, $$\tilde{F}_{0} = {{F_{0} } \mathord{\left/ {\vphantom {{F_{0} } a}} \right. \kern-0pt} a}$$, $$\tilde{E}_{n} = {{E_{n} } \mathord{\left/ {\vphantom {{E_{n} } a}} \right. \kern-0pt} a}$$, and $$\tilde{F}_{n} = {{F_{n} } \mathord{\left/ {\vphantom {{F_{n} } a}} \right. \kern-0pt} a}$$.

Due to the similarity of two elementary problems for an F-F-F-F plate, using coordinate exchange, according to the same rules as presented in Section "Symplectic superposition solutions of C–C–C–C plates", the dimensionless modal displacements $u_{2} \left( {\overline{x},\overline{y}} \right)$ and $v_{2} \left( {\overline{x},\overline{y}} \right)$ can be readily obtained from $v_{1} \left( {\overline{x},\overline{y}} \right)$ and $u_{1} \left( {\overline{x},\overline{y}} \right)$, respectively.

For an F-F-F-F plate, zero in-plane shear stress constraints must be ensured at $x = {0}$, $x = a$, $y = 0$, and $y = b$, i.e.,

24 $$\begin{gathered} \sum\limits_{i = 1}^{2} {\left. {\tau_{xy}^{i} } \right|_{x = 0,a} } = 0 \hfill \\ \sum\limits_{i = 1}^{2} {\left. {\tau_{xy}^{i} } \right|_{y = 0,b} } = 0 \hfill \\ \end{gathered}$$

Under Eq. (24), based on the same procedure as presented in Section "Symplectic superposition solutions of C–C–C–C plates", the natural frequencies and the corresponding vibration modes of the F–F–F–F plate under in-plane vibration are obtained.

## Results

### Comprehensive new natural frequencies and vibration modes

In this section, comprehensive new natural frequencies and vibration modes of isotropic/orthotropic C–C–C–C and F–F–F–F plates are presented so as to provide benchmarks for future comparison. For orthotropic cases, ${{E_{y} } \mathord{\left/ {\vphantom {{E_{y} } {E_{x} = 2.5}}} \right. \kern-0pt} {E_{x} = 2.5}}$ and $\nu_{x} \nu_{y} = \left( {0.3} \right)^{2}$, and the dimensionless frequency parameter is defined as $\Omega_{{{\text{ort}}}} = \omega a\sqrt {{{\rho \left( {1 - \nu_{x} \nu_{y} } \right)} \mathord{\left/ {\vphantom {{\rho \left( {1 - \nu_{x} \nu_{y} } \right)} {E_{x} }}} \right. \kern-0pt} {E_{x} }}}$; for the isotropic cases, $\nu = 0.3$ and the dimensionless frequency parameter is defined as $\Omega_{{{\text{iso}}}} = \omega b\sqrt {{{\rho \left( {1 - \nu^{2} } \right)} \mathord{\left/ {\vphantom {{\rho \left( {1 - \nu^{2} } \right)} E}} \right. \kern-0pt} E}}$.

**Figure 2:**
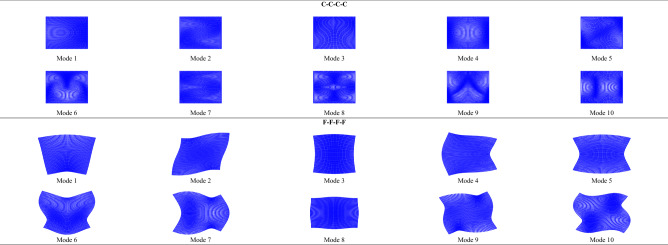
The first ten vibration modes of orthotropic square plates with ${{E_{y} } \mathord{\left/ {\vphantom {{E_{y} } {E_{x} = 2.5}}} \right. \kern-0pt} {E_{x} = 2.5}}$ and $\nu_{x} \nu_{y} = \left( {0.3} \right)^{2}$.

**Figure 3:**
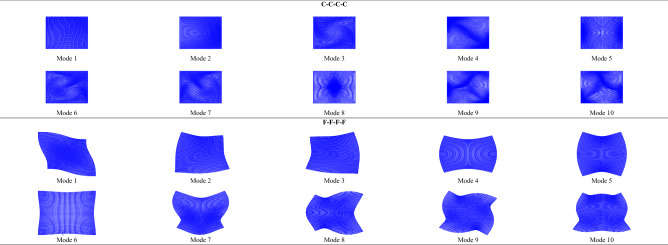
The first ten vibration modes of isotropic square plates with $\nu = 0.3$.

Convergence studies for orthotropic C–C–C–C and F–F–F–F square plates are presented in Table 1. Through verification, 25 series terms ensure the convergence to the last digit of five significant figures of all the results tabulated in this work. In Table 2, the first ten $\Omega_{{{\text{ort}}}}$ of orthotropic C–C–C–C and F–F–F–F plates are respectively provided, each with ${b \mathord{\left/ {\vphantom {b {a = }}} \right. \kern-0pt} {a = }}0.5$, 1, 1.5, and 2. The solutions of orthotropic C–C–C–C plates are validated by the FEM through ABAQUS^26^ with the mesh size being $\left( {{1 \mathord{\left/ {\vphantom {1 {400}}} \right. \kern-0pt} {400}}} \right)a$, the improved Fourier series method in Ref.^7^, and the Ritz method in Ref.^13^, while the solutions of orthotropic F–F–F–F plates are validated by the FEM and the Ritz method in Ref.^13^. In addition, in Table 3, the first ten $\Omega_{{{\text{iso}}}}$ of isotropic C–C–C–C and F–F–F–F plates are respectively provided, each with ${a \mathord{\left/ {\vphantom {a {b = }}} \right. \kern-0pt} {b = }}0.5$, 1, 1.5, and 2. Such solutions are validated by the FEM, the Rayleigh–Ritz method in Ref.^3^, the improved Fourier series method in Ref.^6^, the iterative separation of variables in Ref.^10^, and the function expansion-based method in Ref.^11^. The first ten vibration modes of orthotropic C–C–C–C and F–F–F–F square plates are plotted in Fig. 2, and those of isotropic C–C–C–C and F–F–F–F square plates are plotted in Fig. 3. Such vibration modes have been validated by those obtained by the Rayleigh–Ritz method in Ref.^3^ and the iterative separation of variables in Ref.^10^.

**Table 1:** Convergence study of the first ten frequency parameters ($\Omega_{{{\text{ort}}}}$) of orthotropic square plates with ${{E_{y} } \mathord{\left/ {\vphantom {{E_{y} } {E_{x} = 2.5}}} \right. \kern-0pt} {E_{x} = 2.5}}$ and $\nu_{x} \nu_{y} = \left( {0.3} \right)^{2}$.

Series terms	Modes									
	1st	2nd	3rd	4th	5th	6th	7th	8th	9th	10th
*C–C–C–C*										
5	3.8310	5.2263	5.2846	6.4378	6.7751	7.0871	7.3748	8.4130	8.7117	8.9167
10	**3.8312**	**5.2264**	**5.2850**	6.4384	6.7759	**7.0875**	**7.3752**	**8.4147**	8.7150	8.9172
15	3.8312	5.2264	5.2850	**6.4385**	6.7760	7.0875	7.3752	8.4147	8.7151	8.9172
20	3.8312	5.2264	5.2850	6.4385	6.7760	7.0875	7.3752	8.4147	**8.7152**	**8.9173**
25	3.8312	5.2264	5.2850	6.4385	**6.7761**	7.0875	7.3752	8.4147	8.7152	8.9173
30	3.8312	5.2264	5.2850	6.4385	6.7761	7.0875	7.3752	8.4147	8.7152	8.9173
*F–F–F–F*										
5	2.5812	**2.9059**	**2.9250**	3.5619	**3.7316**	4.5985	**4.7733**	**4.9840**	5.2318	6.0248
10	**2.5811**	2.9059	2.9250	**3.5618**	3.7316	4.5984	4.7733	4.9840	**5.2317**	**6.0234**
15	2.5811	2.9059	2.9250	3.5618	3.7316	**4.5983**	4.7733	4.9840	5.2317	6.0234
20	2.5811	2.9059	2.9250	3.5618	3.7316	4.5983	4.7733	4.9840	5.2317	6.0234
25	2.5811	2.9059	2.9250	3.5618	3.7316	4.5983	4.7733	4.9840	5.2317	6.0234
30	2.5811	2.9059	2.9250	3.5618	3.7316	4.5983	4.7733	4.9840	5.2317	6.0234

Convergent results are in bold.

**Table 2:** The first ten frequency parameters ($\Omega_{{{\text{ort}}}}$) of orthotropic plates with ${{E_{y} } \mathord{\left/ {\vphantom {{E_{y} } {E_{x} = 2.5}}} \right. \kern-0pt} {E_{x} = 2.5}}$ and $\nu_{x} \nu_{y} = \left( {0.3} \right)^{2}$.

${b \mathord{\left/ {\vphantom {b a}} \right. \kern-0pt} a}$	References	Modes									
		1st	2nd	3rd	4th	5th	6th	7th	8th	9th	10th
*C–C–C–C*											
0.5	SSM	5.5661	7.6283	9.3270	9.5563	10.137	10.497	10.531	11.936	12.071	12.754
	FEM	5.5683	7.6328	9.3420	9.5666	10.148	10.510	10.541	11.952	12.090	12.780
	Ref.^7^	5.5660	7.6304	9.3294	9.5574	10.141	10.499	10.532	11.941	12.078	12.769
	Ref.^13^	5.5660	7.6284	9.3270	9.5564	10.137	10.497	10.531	11.936	12.071	12.754
1	SSM	3.8312	5.2264	5.2850	6.4385	6.7761	7.0875	7.3752	8.4147	8.7152	8.9173
	FEM	3.8326	5.2322	5.2875	6.4470	6.7834	7.0996	7.3946	8.4343	8.7351	8.9428
	Ref.^7^	3.8314	5.2280	5.2852	6.4408	6.7772	7.0898	7.3786	8.4190	8.7198	8.9222
	Ref.^13^	3.8312	5.2264	5.2850	6.4384						
1.5	SSM	3.4239	3.9382	4.1144	5.2986	5.7013	5.9833	6.3903	6.7086	6.7582	7.4814
	FEM	3.4257	3.9400	4.1181	5.3078	5.7106	5.9948	6.4062	6.7298	6.7704	7.4956
	Ref.^7^	3.4240	3.9384	4.1154	5.3010	5.7028	5.9856	6.3928	6.7164	6.7610	7.4846
2	SSM	3.2771	3.3344	3.6674	4.3852	5.1319	5.1520	5.4524	6.0679	6.2422	6.3512
	FEM	3.2787	3.3357	3.6700	4.3896	5.1368	5.1609	5.4617	6.0817	6.2584	6.3639
	Ref.^7^	3.2772	3.3346	3.6682	4.3868	5.1338	5.1554	5.4562	6.0704	6.2496	6.3536
	Ref.^13^	3.2770	3.3344	3.6674	4.3852	5.1320	5.1520	5.4524	6.0678	6.2422	6.3512
*F–F–F–F*											
0.5	SSM	2.0293	2.9846	3.7660	5.5983	5.6513	5.8503	7.2524	7.2927	7.7188	7.8381
	FEM	2.0298	2.9850	3.7688	5.6041	5.6560	5.8531	7.2614	7.3013	7.7325	7.8487
	Ref.^13^	2.0294	2.9846	3.766	5.5982						
1	SSM	2.5811	2.9059	2.9250	3.5618	3.7316	4.5983	4.7733	4.9840	5.2317	6.0234
	FEM	2.5829	2.9076	2.9260	3.5652	3.7334	4.6042	4.7777	4.9859	5.2435	6.0334
	Ref.^13^	2.5812	2.9058	2.925	3.5618						
1.5	SSM	2.1502	2.4499	2.6934	2.7690	3.0338	3.5638	3.7315	3.8868	4.0018	5.0016
	FEM	2.1517	2.4516	2.6944	2.7712	3.0351	3.5652	3.7357	3.8907	4.0100	5.0117
2	SSM	1.4516	2.1543	2.2554	2.8392	2.8482	3.0867	3.1875	3.2193	3.2253	4.0238
	FEM	1.4521	2.1557	2.2559	2.8412	2.8498	3.0904	3.1896	3.2210	3.2279	4.0284
	Ref.^13^	1.4516	2.1544	2.2554	2.8392

**Table 3:** The first ten frequency parameters ($\Omega_{{{\text{iso}}}}$) of isotropic plates with $\nu = 0.3$.

${a \mathord{\left/ {\vphantom {a b}} \right. \kern-0pt} b}$	References	Modes									
		1st	2nd	3rd	4th	5th	6th	7th	8th	9th	10th
*C–C–C–C*											
1	SSM	3.5552	3.5552	4.2350	5.1857	5.8586	5.8944	5.8944	6.7077	7.1132	7.1132
	FEM	3.5566	3.5566	4.2394	5.1912	5.8676	5.9081	5.9081	6.7168	7.1282	7.1282
	Ref.^3^	3.555	3.555	4.235	5.186	5.859	5.895				
	Ref.^6^	3.554	3.554	4.236	5.185	5.859	5.896				
	Ref.^10^	3.5588	3.5588	4.2376	5.2293	5.8657	5.9233	5.9233			
	Ref.^11^	3.5552	3.5552	4.2350	5.1857	5.8586	5.8944	5.8944	6.7077		
1.5	SSM	4.1127	4.9252	5.4025	6.5644	6.6164	6.6164	8.1996	8.3581	8.4290	8.9437
	FEM	4.1144	4.9278	5.4085	6.5714	6.6123	6.6269	8.2236	8.3869	8.4451	8.9316
	Ref.^6^	4.112	4.923	5.402	6.564	6.602	6.617				
2	SSM	4.7890	6.3786	6.7121	7.0487	7.6083	8.1402	8.9980	9.5156	9.7165	10.601
	FEM	4.7908	6.3823	6.7182	7.0532	7.6163	8.1536	9.0122	9.5290	9.7337	10.626
	Ref.^3^	4.789	6.379	6.712	7.049	7.608	8.140				
	Ref.^6^	4.788	6.374	6.710	7.048	7.608	8.140				
	Ref.^10^	4.7903	6.3872	6.7184	7.0535	7.6310	8.2172	9.0716			
*F–F–F–F*											
1	SSM	2.3194	2.4722	2.4722	2.6271	2.9859	3.4505	3.7214	3.7214	4.3009	4.9666
	FEM	2.3219	2.4736	2.4736	2.6293	2.9884	3.4537	3.7272	3.7272	4.3133	4.9795
	Ref.^3^	2.321	2.472	2.472	2.628	2.987	3.452				
	Ref.^6^	2.321	2.472	2.472	2.629	2.988	3.452				
1.5	SSM	2.1964	2.8809	2.9148	3.9375	3.9708	4.3804	4.5286	4.5935	4.7360	6.1404
	FEM	2.1976	2.8829	2.9155	3.9403	3.9750	4.3851	4.5325	4.6023	4.7387	5.8893
	Ref.^6^	2.197	2.881	2.915	3.938	3.971	4.380				
2	SSM	1.9537	2.9608	3.2671	4.7263	4.7841	5.2045	5.2045	5.3651	6.1466	6.4475
	FEM	1.9542	2.9611	3.2694	4.7313	4.7885	5.2090	5.2596	5.3703	6.1506	6.4605
	Ref.^3^	1.954	2.961	3.267	4.726	4.784	5.205				
	Ref.^6^	1.954	2.961	3.268	4.725	4.785	5.205

**Figure 4:**
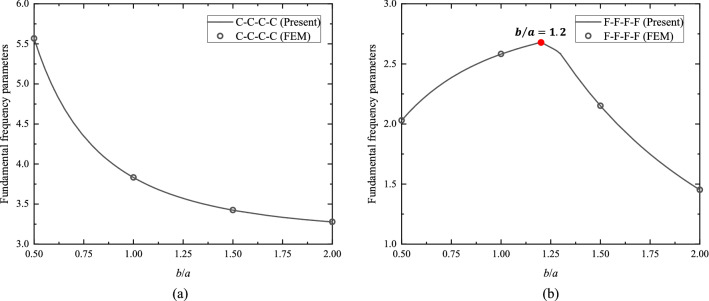
Fundamental frequency parameters ($\Omega_{{{\text{ort}}}}$) versus ${b \mathord{\left/ {\vphantom {b a}} \right. \kern-0pt} a}$ for (**a**) orthotropic C–C–C–C plates and (**b**) orthotropic F–F–F–F plates, with ${{E_{y} } \mathord{\left/ {\vphantom {{E_{y} } {E_{x} = 2.5}}} \right. \kern-0pt} {E_{x} = 2.5}}$ and $\nu_{x} \nu_{y} = \left( {0.3} \right)^{2}$.

**Figure 5:**
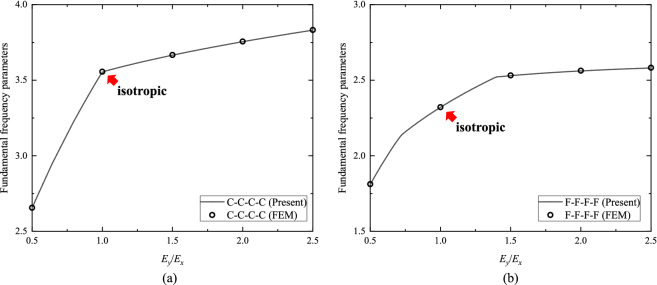
Fundamental frequency parameters ($\Omega_{{{\text{ort}}}}$) versus ${{E_{y} } \mathord{\left/ {\vphantom {{E_{y} } E}} \right. \kern-0pt} E}_{x}$ for (**a**) orthotropic C–C–C–C square plates and (**b**) orthotropic F–F–F–F square plates, with $\nu_{x} \nu_{y} = \left( {0.3} \right)^{2}$.

With the accurate analytic solutions at hand, quantitative parametric analyses are readily conducted. Figure 4a,b plot the fundamental $\Omega_{{{\text{ort}}}}$ versus ${b \mathord{\left/ {\vphantom {b a}} \right. \kern-0pt} a}$, ranging from 0.5 to 2, for orthotropic C–C–C–C and F–F–F–F plates, respectively, with scattered FEM results, indicated by the circles, added for comparison. The fundamental $\Omega_{{{\text{ort}}}}$ of orthotropic C–C–C–C plates decrease with the increase of ${b \mathord{\left/ {\vphantom {b a}} \right. \kern-0pt} a}$. For orthotropic F–F–F–F plates, however, it is found that the fundamental $\Omega_{{{\text{ort}}}}$ increase with the increase of ${b \mathord{\left/ {\vphantom {b a}} \right. \kern-0pt} a}$ at first and then decrease, with the maximum achieved at ${b \mathord{\left/ {\vphantom {b a}} \right. \kern-0pt} a} = 1.2$ as indicated by the red dot. Moreover, Fig. 5a, b illustrate the fundamental $\Omega_{{{\text{ort}}}}$ versus ${{E_{y} } \mathord{\left/ {\vphantom {{E_{y} } {E_{x} }}} \right. \kern-0pt} {E_{x} }}$, ranging from 0.5 to 2.5, for orthotropic C–C–C–C and F–F–F–F square plates, respectively, where the FEM results are also added for comparison. For orthotropic C–C–C–C square plates, it is observed that the fundamental $\Omega_{{{\text{ort}}}}$ increase when ${{E_{y} } \mathord{\left/ {\vphantom {{E_{y} } {E_{x} }}} \right. \kern-0pt} {E_{x} }}$ becomes larger, but the growth rate has a sudden change after ${{E_{y} } \mathord{\left/ {\vphantom {{E_{y} } {E_{x} }}} \right. \kern-0pt} {E_{x} }} = 1$, i.e., the isotropic case. For orthotropic F–F–F–F square plates, the fundamental $\Omega_{{{\text{ort}}}}$ also increase with the increase of ${{E_{y} } \mathord{\left/ {\vphantom {{E_{y} } {E_{x} }}} \right. \kern-0pt} {E_{x} }}$; nevertheless, the growth rate does not vary rapidly at ${{E_{y} } \mathord{\left/ {\vphantom {{E_{y} } {E_{x} }}} \right. \kern-0pt} {E_{x} }} = 1$. Besides the present parametric analyses, the effects of any other parameters of interest on in-plane vibration of plates can be investigated with the obtained analytic solutions.

## Concluding remarks

In this paper, we have presented new analytic free in-plane vibration solutions for isotropic/orthotropic C–C–C–C and F–F–F–F plates by extending the SSM that has never been applied to in-plane elasticity problems in any existing works. The main solution procedure of the SSM can be summarized as: (1) establishing the governing equation in the Hamiltonian system; (2) utilizing the separation of variables and the symplectic eigen expansion to yield the analytic solutions of two elementary problems; (3) superposition of the elementary problems’ solutions giving the final analytic free in-plane vibration solutions. Such a solution procedure yields the analytic solutions that can satisfy both the governing partial differential equations and non-Lévy-type boundary conditions for in-plane vibration. Rational and rigorous solution procedure, fast convergence, and high accuracy of the proposed SSM-based framework are well validated, indicating the capability of all tabulated results to serve as benchmarks for related studies. In the light of the advantages of the SSM-based framework, more analytic results of complicated in-plane elasticity analysis can be explored in our future studies.

## Figures and Tables

**Figure 6:**
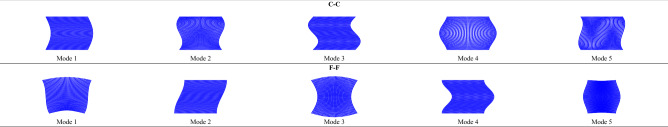
The first five vibration modes of isotropic plates with two opposite SS1 edges at $x = {0}$ and $x = a$ (${b \mathord{\left/ {\vphantom {b a}} \right. \kern-0pt} a} = 1.2$ and $\nu = 0.3$).

**Figure 7:**
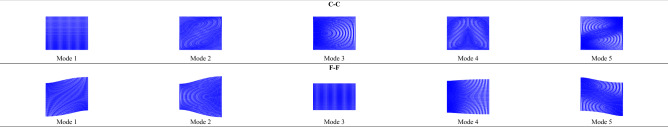
The first five vibration modes of isotropic plates with two opposite SS2 edges at $x = {0}$ and $x = a$ (${b \mathord{\left/ {\vphantom {b a}} \right. \kern-0pt} a} = 1.2$ and $\nu = 0.3$).

## Data Availability

All data generated or analyzed during this study are included in this published article.

## Appendix 1: Detailed expressions of $k_{1 \sim 6}$

$$k_{1}\left( {\mu} \right) = \frac{{\eta_{3} \lambda_{1} \mu }}{{\eta_{1} \lambda_{1}^{2} + \mu^{2} + R^{2} }}$$

$$k_{2}\left( {\mu} \right) = \frac{{\eta_{3} \lambda_{2} \mu }}{{\eta_{1} \lambda_{2}^{2} + \mu^{2} + R^{2} }}$$

$$k_{3}\left( {\mu} \right) = A_{66} \left[ {k_{1} \lambda_{1} \left( {1 - \eta_{3} } \right) + \mu \eta_{2} } \right]$$

$$k_{4}\left( {\mu} \right) = A_{66} \left[ {k_{2} \lambda_{2} \left( {1 - \eta_{3} } \right) + \mu \eta_{2} } \right]$$

$$k_{5}\left( {\mu} \right) = A_{66} \left( {k_{1} \mu - \lambda_{1} } \right)$$

$$k_{6}\left( {\mu} \right) = A_{66} \left( {k_{2} \mu - \lambda_{2} } \right)$$

## Appendix 2: Frequency results of some representative Lévy-type plates

In this Appendix, natural frequencies (Table

**Table 4:** The first five frequency parameters ($\Omega_{{{\text{iso}}}}$) of representative Lévy-type isotropic plates with ${b \mathord{\left/ {\vphantom {b a}} \right. \kern-0pt} a} = 1.2$ and $\nu = 0.3$.

$y = 0$ and $y = b$	References	Modes				
		1st	2nd	3rd	4th	5th
*Two opposite SS1 edges at* $x = {0}$ *and* $x = a$						
C–C	SSM	1.8586	3.4359	3.7172	3.9802	5.0166
	Ref.^7^	1.8585	3.4362	3.7184	3.9802	5.0180
	Ref.^9^	1.8585	3.4360	3.7173	3.9802	5.0166
F–F	SSM	1.7784	1.8586	2.8438	3.7172	3.8549
	Ref.^7^	1.7784	1.8585	2.8439	3.7170	3.8551
	Ref.^9^	1.7784	1.8585	2.8439	3.7173	3.8549
*Two opposite SS2 edges at* $x = {0}$ *and* $x = a$						
C–C	SSM	3.1416	3.4359	3.9802	5.0166	5.3337
	Ref.^7^	3.1414	3.4360	3.9802	5.0171	5.3342
	Ref.^9^	3.1416	3.4360	3.9802	5.0166	5.3338
F–F	SSM	1.7784	2.8438	3.1416	3.8549	3.9254
	Ref.^7^	1.7782	2.8439	3.1416	3.8546	3.9253
	Ref.^9^	1.7784	2.8439	3.1416	3.8549	3.9253

^4^) and vibration modes (Figs. 6 and 7) of some representative Lévy-type plates are presented. By directly adopting the analytic solutions in Eqs. (15) and (18), such frequency results are obtained. An interesting phenomenon is also observed in Table 4. The frequency parameters of the second, fourth, and fifth vibration modes of the plate with two SS1 opposite edges at $x = {0}$ and $x = a$ and the other two edges clamped (SS1–C–SS1–C) are the same as those of the second, third, and fourth vibration modes of the plate with two SS2 opposite edges at $x = {0}$ and $x = a$ and the other two edges clamped (SS2–C–SS2–C); those of the first, third, and fifth vibration modes of the plate with two SS1 opposite edges at $x = {0}$ and $x = a$ and the other two edges free (SS1–F–SS1–F) are the same as those of the first, second, and fourth vibration modes of the plate with two SS2 opposite edges at $x = {0}$ and $x = a$ and the other two edges free (SS2–F–SS2–F); those of the first and third vibration modes of the SS1–C–SS1–C plate are the same as those of the second and fourth vibration modes of the SS1–F–SS1–F plate; the frequency parameter of the first vibration mode of the SS2–C–SS2–C plate is the same as that of the third vibration mode of the SS2–F–SS2–F plate.
